# Inulin Supplementation Does Not Reduce Plasma Trimethylamine *N*-Oxide Concentrations in Individuals at Risk for Type 2 Diabetes

**DOI:** 10.3390/nu10060793

**Published:** 2018-06-20

**Authors:** Mary Elizabeth Baugh, Cortney N. Steele, Christopher J. Angiletta, Cassie M. Mitchell, Andrew P. Neilson, Brenda M. Davy, Matthew W. Hulver, Kevin P. Davy

**Affiliations:** 1 Department of Human Nutrition, Foods, and Exercise, 295 West Campus Dr., Virginia Tech, Blacksburg, VA 24061, USA; mebaugh@vt.edu (M.E.B.); cnsteele@vt.edu (C.N.S.); chrisa88@vt.edu (C.J.A.); casmitch@vt.edu (C.M.M.); bdavy@vt.edu (B.M.D.); hulvermw@vt.edu (M.W.H.); 2 Translational Obesity Research Graduate Education Program, Virginia Tech, Blacksburg, VA 24061, USA; andrewn@vt.edu; 3 Department of Food Science and Technology, Integrated Life Sciences Building, 1981 Kraft Dr., Blacksburg, VA 24060, USA; 4 The Metabolic Phenotyping Core, 1981 Kraft Dr., Virginia Tech, Blacksburg, VA 24061, USA

**Keywords:** prediabetes, prebiotic, metabolite, cardiovascular, metabolism

## Abstract

Trimethylamine *N*-oxide (TMAO) is associated with type 2 diabetes (T2DM) and increased risk of adverse cardiovascular events. Prebiotic supplementation has been purported to reduce TMAO production, but whether prebiotics reduce fasting or postprandial TMAO levels is unclear. Sedentary, overweight/obese adults at risk for T2DM (*n* = 18) were randomized to consume a standardized diet (55% carbohydrate, 30% fat) with 10 g/day of either an inulin supplement or maltodextrin placebo for 6 weeks. Blood samples were obtained in the fasting state and hourly during a 4-h high-fat challenge meal (820 kcal; 25% carbohydrate, 63% fat; 317.4 mg choline, 62.5 mg betaine, 8.1 mg l-carnitine) before and after the diet. Plasma TMAO and trimethylamine (TMA) moieties (choline, l-carnitine, betaine, and γ-butyrobetaine) were measured using isocratic ultraperformance liquid chromatography-tandem mass spectrometry (UPLC-MS/MS). There were no differences in fasting or postprandial TMAO or TMA moieties between the inulin and placebo groups at baseline (all *p* > 0.05). There were no significant changes in fasting or postprandial plasma TMAO or TMA moiety concentrations following inulin or placebo. These findings suggest that inulin supplementation for 6 weeks did not reduce fasting or postprandial TMAO in individuals at risk for T2DM. Future studies are needed to identify efficacious interventions that reduce plasma TMAO concentrations.

## 1. Introduction

Recent estimates indicate that the prevalence of prediabetes is increasing worldwide and the number people suffering from type 2 diabetes (T2DM) will likely exceed 470 million by 2030 [1]. Importantly, prediabetes is associated with a high risk for both development of T2DM as well as adverse cardiovascular disease (CVD)-related events [2,3]. As such, effective early intervention is needed to mitigate cardiometabolic risk in individuals at risk for developing T2DM.

The gut microbiota has been implicated in the pathogenesis of T2DM and CVD. Recently, trimethylamine *N*-oxide (TMAO), a metabolite originating from gut microbial metabolism of dietary trimethylamine (TMA)-containing moieties, has been reported to be elevated in T2DM and predictive of future cardiovascular events in several independent cohorts [4,5,6,7,8]. Gut bacteria releases TMA from primarily dietary choline, but also l-carnitine and betaine. TMA enters the circulation and is oxidized by hepatic flavin-containing monooxygenase 3 to form TMAO [5,6,7,9].

There are presently no efficacious interventions for reducing plasma TMAO concentrations in humans. Prebiotics and probiotics have been suggested as strategies that may be efficacious in reducing TMA synthetic capacity by modulating gut microbiota composition [10,11]. To date, the available evidence does not support the use of probiotics to reduce plasma TMAO concentrations [12,13]. However, whether prebiotics reduce fasting or postprandial TMAO in any population is unclear. Accordingly, we tested the hypothesis that inulin, a prebiotic fiber from chicory root, would reduce plasma TMAO concentrations in middle-aged and older adults at risk for T2DM.

## 2. Materials and Methods

### 2.1. Participants

Eighteen overweight or obese (body mass index; BMI 25–40 kg/m^2^) men and women aged 40–75 years at increased risk for T2DM that participated in a larger study comprised the sample for the present study. Details of participant characteristics and the study design have been published previously [14] (Clinicaltrials.gov Identifier: NCT02346838). Briefly, participants were weight-stable (±2.5 kg) for ≥6 months and sedentary to recreationally active (≤2 days/week and <20 min/day of low intensity activity for the previous 12 months), with blood pressure ≤160/100 mm Hg and total cholesterol values ≤300 mg/dL and triglyceride values of ≤450 mg/dL. In addition, participants were prediabetic or at increased risk of developing diabetes (American Diabetes Association risk assessment score ≥5, glycated hemoglobin (HbA1C) value of 5.7–6.4%, with a fasting blood glucose value of 100–125 mg/dL, or 2-h oral glucose tolerance test value of 140–200 mg/dL). Participants had not taken antibiotics for ≥3 months prior to study participation. They also were not taking medications or supplements (e.g., prebiotics/probiotics, fiber supplements) that could affect dependent variables and were free from overt chronic diseases as assessed by blood chemistry, urinalysis, and a health history questionnaire. The Virginia Polytechnic Institute and State University Institutional Review Board approved the study protocol (#13-694), and written and verbal informed consent were obtained from each participant. 

### 2.2. Experimental Design

A randomized double-blind, placebo-controlled, parallel group design was used for this study. After completing baseline measures, participants were stratified by sex and randomized into one of two groups: supplementation with 10 g/day of inulin derived from chicory root (Frutafit^®^ IQ, Sensus American, Inc., Lawrenceville, NJ, USA; 2 kcal/g) or with 10 g/day of a placebo (maltodextrin; 4 kcal/g) for 6 weeks. Participants consumed the supplement mixed in water with their supervised breakfast meal. For the first 7 days, participants consumed either 5 g/day of inulin or placebo and subsequently moved to 10 g/day to minimize any potential gastrointestinal symptoms that could occur with a sudden increase in prebiotic consumption. Measurements of key outcome variables were performed at baseline and following the intervention period.

### 2.3. Controlled Feeding

All diets were controlled and standardized to minimize potential effects of inter-individual variability in habitual dietary intake on dependent variables. Energy requirements were estimated for each individual using the Mifflin-St. Jeor equation based on age, weight, height, and sex [15]. All participants consumed a standardized diet (55% carbohydrate, 30% fat (8% saturated fat), 15% protein, ≤8 g/1000 kcal total dietary fiber, ≤2 g/1000 kcal soluble fiber) isocaloric to their estimated energy requirements for 6 weeks. A 7-day cycle menu was created for energy intake levels between 1500 and 3000 kcals/day in 500-kcal increments using Nutrition Data Systems for Research software (NDS-R, v. 2014; University of Minnesota, Minneapolis, MN, USA). In addition, 250-kcal snack modules of the same macronutrient composition as the standardized diet were added or subtracted to each participant’s daily food allotment if weight changed >1.36 kg (3 lbs) daily. Probiotic foods (e.g., yogurt) were not included in the menus. Participants were allowed to consume 450 g/day (~16 fl. oz./day) caffeinated black coffee or unsweetened black tea if they reported regularly consuming either. 

Participants reported to our metabolic kitchen each weekday to consume breakfast and the supplement drink and to pick up a cooler with food for the remainder of the day. Participants were instructed to consume only foods and beverages (except water) provided from the metabolic kitchen and to report all non-study foods consumed to research study staff to monitor compliance. Dietary compliance was objectively assessed by weighing each food item provided in each cooler and subtracting out empty container weights after coolers were returned. Body weight was measured on a digital scale (Scale-Tronix Model 5002, Welch Allyn, Skaneateles Falls, NY, USA) each time participants reported to metabolic kitchen to pick up food to ensure weight stability.

### 2.4. Procedures

Baseline body weight and height was measured with a digital scale with a stadiometer (Scale-Tronix Model 5002). Brachial arterial pressure was measured in a seated position using automated sphygmomanometry (Press-Mate BP 8800, Colin Medical Instruments Corp, San Antonio, TX, USA). Habitual dietary intake was assessed using 4-day food intake records. Participants were given detailed verbal and written instructions about how to properly record intake of foods and beverages for 4 consecutive days (3 weekdays and 1 weekend day). Participants were also given a two-dimensional food model booklet to assist in determining accurate amounts of consumed foods and were asked to bring in any food labels that may assist in identifying the exact food consumed. Returned food records were reviewed with the participant by a trained diet technician using a multiple-pass method adapted from techniques utilized in 24-h recalls [16]. Habitual dietary intakes were assessed by a diet technician using NDS-R software (v. 2014; University of Minnesota).

Daily choline and betaine intake were assessed for both habitual and the standardized diets by averaging the nutrient totals report output in NDS-R. Daily l-carnitine intake was assessed by determining the appropriate food group classification and serving amount in NDS-R and then converting these servings into grams. Published conversion tables were then used to convert gram servings of each food into milligram intake of l-carnitine [17,18].

### 2.5. Fasting and Postprandial Blood Collection

All measurements took place at the Human Integrative Physiology Laboratory between the hours of 5:00 and 11:00 a.m. Participants were fasted for the prior 12 h, performed no vigorous physical activity for the prior 36 h, and were free from acute illness for the prior 2 weeks. All participants consumed a high-fat meal of two sausage, egg, and cheese breakfast biscuits (820 kcal; 63% total fat (26% saturated fat), 25% carbohydrate, and 12% protein; 314 mg choline, 63 mg betaine, and 8 mg l-carnitine) before and after the 6-week standardized diet to assess meal- and diet-effect on plasma TMAO concentrations. Blood draws were obtained in the fasting state and each hour for four hours after consumption of the breakfast meal. Blood samples were centrifuged, and plasma was stored at −80°C until analysis. We previously used this approach in a prior study [12] indicating that postprandial TMAO concentrations increased following 4 weeks of a consuming of high-fat diet. 

### 2.6. Mass Spectrometry

Plasma TMAO, choline, betaine, l-carnitine, and γ-butyrobetaine were measured according to previously described methods [12,19]. Plasma samples were thawed at room temperature. A stock internal standard (IS) solution was prepared by diluting 1 mL of aqueous choline chloride-d_9_ (25 μM, Sigma, St. Louis, MO, USA), betaine-d_9_, TMAO-d_9_ (25 μM, Cambridge Isotope Laboratories, Tewksbury, MA, USA), and l-carnitine-d_9_ (120 μM, Cambridge Isotope Laboratories, Tewksbury, MA, USA) to a final volume of 100 mL with acetonitrile (ACN). Plasma (25 μL) and IS/ACN solution (300 μL) were combined, vortexed, and centrifuged (17,000× *g*, 3 min, room temperature). Supernatants were syringe filtered (polytetrafluoroethylene, 4 mm, 0.2 μm pore size) into certified Waters liquid chromatography mass spectrometry vials with spring-loaded deactivated glass inserts (150 μL) and analyzed immediately. Samples were analyzed (5 μL) on a Waters Acquity isocratic ultraperformance liquid chromatography-tandem mass spectrometry (UPLC-MS/MS) instrument (Milford, MA, USA). Separations were performed on a Waters BEH HILIC column (2.1 × 100 mm; 1.7 μm particle size) with a BEH HILIC VanGuard pre-column (2.1 × 5 mm; 1.7 μm). Column and sample temperatures were 30 and 10 °C, respectively. The mobile phases were 15 mM ammonium formate, pH 3.5, (phase A) and ACN (phase B). The flow rate was 0.65 mL/min, and isocratic elution was achieved (20% A/80% B) over 3 min. Following separation, analytes and ISs were quantified using electrospray ionization in (+)-mode. Source and capillary temperatures were 150 and 400 °C, respectively. Capillary voltage was 0.60 kV, and desolvation and cone gas (both N_2_) flow rates were 800 and 20 L/h, respectively. Collision-induced dissociation was performed using argon as the collision gas. Compounds were quantified using optimized multi-reaction monitoring (MRM) functions shown in Table 1. MRMs were optimized to achieve 12 points/10 s peak, and the detection span was ±0.2 atomic mass units. Quantification was performed using ratio of the target analyte and respective IS peak areas, based on authentic external standard curves prepared using a wide range of target analyte concentrations (choline chloride, TMAO, betaine, l-carnitine, and γ-butyrobetaine hydrochloride; Sigma, St. Louis, MO, USA) bracketing the peak areas observed in the plasma samples and the same IS concentrations used to prepare the plasma samples. 

### 2.7. Statistical Analyses

Statistical analyses were conducted using SPSS statistical software (version 24, 2016; IBM, Armonk, NY, USA), and area under the curve (AUC) for each postprandial analyte was calculated with Excel (version 15.32 for Mac, 2017; Microsoft, Redmond, WA, USA) using the trapezoidal rule. Prism (version 7 for Mac OS X, 2017; GraphPad Software, La Jolla, CA, USA) was used to generate figures. Independent samples t-tests were used to compare group baseline characteristics. Two-way repeated-measures analysis of variance was used to assess effects of the intervention, high fat meal, and intervention and meal interaction on the dependent variables. Pearson product-moment correlations were used to determine relationships among variables. Data not normally distributed were transformed and analyzed. However, raw values are presented because transforming the data did not alter the results. The significance level of *p* < 0.05 was set a priori for all statistical tests. 

## 3. Results

### 3.1. Participant Characteristics

Baseline participant characteristics are shown in Table 2. There were no significant differences between intervention groups at baseline in BMI, body fat percentage, fasting plasma glucose, 2-h oral glucose tolerance, or plasma lipids and lipoproteins. In addition, there was no significant difference in sex distribution between groups. There was no significant difference between intervention groups in body weight change over time. 

### 3.2. Dietary Intake

Habitual dietary intake is shown in Table 3. The inulin group reported significantly lower habitual dietary sodium and choline intake compared with the placebo group. The inulin group also reported significantly lower habitual dietary protein intake in total grams; however, as a percentage of total energy intake, habitual dietary protein intake was not different between groups (*p* > 0.05). Dietary intake in the standardized diet was similar between groups (Table S1). The dietary composition of each standardized diet caloric level is shown in Table S2. Overall compliance with the standardized diet was 97.5%.

### 3.3. Fasting and Postprandial Plasma TMAO and TMA Moiety Concentrations

**Figure 1:**
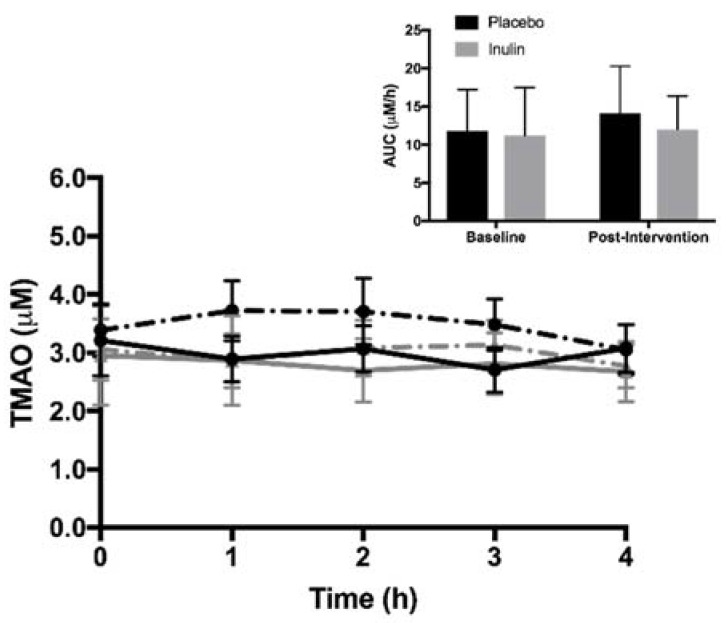
Fasting and postprandial trimethylamine *N*-oxide (TMAO) concentrations. Inset: Area under the curve (AUC) between placebo and inulin groups. Values are expressed as mean ± standard error of the mean. 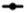 placebo at baseline, 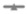 inulin at baseline, 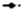 placebo post-intervention, 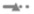 inulin post-intervention.

**Figure 2:**
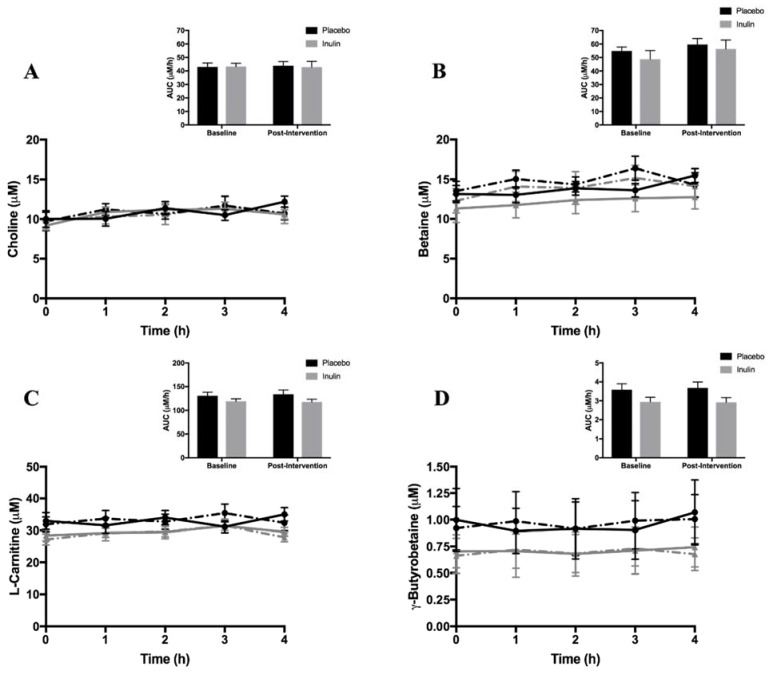
Fasting and postprandial plasma (**A**) choline, (**B**) betaine, (**C**) l-carnitine, and (**D**) γ-butyrobetaine concentrations at baseline and post-intervention. Insets: Areas under the curve (AUC) between placebo and inulin groups for (**A**) choline, (**B**) betaine, (**C**) l-carnitine, and (**D**) γ-butyrobetaine. 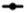 placebo at baseline, 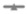 inulin at baseline, 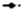 placebo post-intervention, 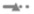 inulin post-intervention.

Fasting and postprandial plasma TMAO concentrations are shown in Figure 1. There were no differences (*p* = 0.80) in fasting plasma TMAO concentrations between the groups at baseline. Fasting and postprandial TMAO concentrations did not significantly change in the inulin or placebo groups. Similarly, plasma TMAO concentration AUC did not change following the intervention in the inulin and placebo groups (*p* = 0.777 and *p* = 0.310, respectively). There were no differences in fasting concentrations of choline, betaine, l-carnitine, and γ-butyrobetaine (all *p* > 0.05) between the inulin and placebo groups at baseline or in response to the intervention (Figure 2). Likewise, plasma concentrations of all AUCs for TMA moieties did not change significantly in either group following intervention.

### 3.4. Correlations

Habitual dietary choline intake was correlated with baseline fasting plasma γ-butyrobetaine concentrations (*r* = 0.773, *p* < 0.001); however, habitual dietary betaine and l-carnitine intakes were not correlated with baseline fasting plasma concentrations of any TMA moiety. Fasting plasma choline concentrations were correlated with fasting plasma TMAO concentrations at baseline (*r* = 0.664, *p* = 0.003).

## 4. Discussion

The major new finding of the present preliminary study is that inulin supplementation did not reduce fasting or postprandial plasma TMAO concentrations in individuals at risk for developing T2DM. We previously reported that a multi-species probiotic (VSL#3) did not attenuate the increase in TMAO following a high fat diet in young men [12]. Taken together, these findings suggest that other approaches are needed to target gut microbiota-generated increases in TMAO and associated cardiometabolic risk in humans.

Reducing intake of dietary sources of TMA moieties, such as choline and l-carnitine, are a logical approach to reducing TMAO concentrations. In this regard, TMAO production is lower in vegans/vegetarians [5,20] and those who demonstrate high adherence to a Mediterranean dietary pattern [21] compared with omnivores; lower plasma TMAO concentrations are sometimes [5] but not always observed in vegans [22]. As such, limiting dietary sources of TMA moieties may be a reasonable strategy for reducing TMAO concentrations. However, it is important to emphasize that choline is an essential nutrient for humans and prolonged dietary deficiency may lead to non-alcoholic fatty liver disease and cognitive decline [23]. Future studies will be necessary to test the feasibility of such an approach with the latter in mind.

Recently, Wang et al. [24] identified a small molecule (3,3-dimethyl-1-butanol (DMB)) that is a non-lethal gut microbial enzyme inhibitor that reduces TMAO and attenuates foam cell formation and atherosclerosis in a preclinical model. Unfortunately, DMB is not available for use in humans. Nevertheless, such an approach that targets specific gut microbial enzymes involved in TMA synthesis is a particularly attractive therapeutic approach in need of additional testing and development, particularly in humans.

Bergeron et al. [25] reported that plasma TMAO concentrations were increased following two weeks of consuming a high resistant starch diet in overweight and obese adults. However, the latter was only observed when carbohydrate intake was low (dietary fat intake was also higher, ~40%). In this regard, we have previously demonstrated that postprandial TMAO concentrations were increased following 5-days of consuming a high fat diet [19]. The possibility that fasting TMAO concentrations might also increase if the high fat diet were extended in duration cannot be excluded. Nevertheless, these prior observations highlight the need for controlling food intake such we have done in the present study.

The present study is the first to use controlled feeding of any duration with high dietary compliance and documented weight stability to address this influence of inulin supplementation on plasma TMAO concentrations in humans. 

There were some limitations that should be acknowledged. First, our findings are limited to a small sample of adults at risk for T2DM and, as such, our study may not have been adequately powered or generalizable to other populations. 

Second, our sample of participants had mean plasma TMAO concentrations that have been associated with relatively lower risk of major adverse cardiovascular events [7]. As such, our ability to intervene may have been limited. However, the middle-aged and older adults at risk of developing T2DM in the present study had plasma TMAO concentrations approximately 2–3-fold higher than we have previously reported in healthy young men (~3–4 uM vs. ~1–2 uM, respectively) [12,19]. As such, we believe that there was a reasonable opportunity to intervene in the mild to modest elevations in plasma TMAO concentrations observed in the present study. Importantly, we should emphasize that the intake of choline and l-carnitine in our standardized diet were less than the usual intake of 340 mg/day [26] and 85–215 mg/day (0.96–2.4 mg/kg body weight/day) [27], respectively, in the U.S. population. Future studies that control dietary intake will need to consider this issue. 

Third, our intervention was only 6 weeks in duration and we did not provide gut microbiome data to document a bifidogenic effect of inulin supplementation in the present study. However, we should emphasize that inulin is considered to be among the most studied and well-established prebiotics [28,29]. Importantly, a significant increase in the abundance of bifidobacteria has been observed in healthy adults with inulin supplementation at doses as low as 2.5 g/day for 2 weeks [30]. Nonetheless, we cannot exclude the possibility that a higher dose or longer duration of inulin supplementation may have produced a different outcome.

## 5. Conclusions

In summary, the findings of our preliminary investigation suggest that six weeks of inulin supplementation did not reduce fasting or postprandial plasma TMAO concentrations in adults at risk for T2DM. Future studies are needed to identify efficacious interventions to lower plasma TMAO.

## Figures and Tables

**Table 1:** Multi-reaction monitoring settings for isocratic ultraperformance liquid chromatography-tandem mass spectrometry detection. Abbreviations: TMAO, trimethylamine *N*-oxide.

Compound	Retention Time (min)	Parent [M + H]^+^ (*m*/*z*)	Daughter (*m*/*z*)	Cone Voltage (V)	Collision Energy (eV)
Betaine	1.25	117.15	118.24	59	44
γ-Butyrobetaine	0.98	146.26	87.00	26	16
Betaine-d_9_	1.25	126.14	127.3	68	46
Choline	1.13	103.16	104.2	60	38
Choline-d_9_	1.11	112.16	113.32	69	40
TMAO	2.01	75.11	76.16	59	40
TMAO-d_9_	1.98	84.12	85.22	68	40

**Table 2:** Participant characteristics at baseline.

Characteristic	Placebo *n* = 11 (6 Females)	Inulin *n* = 7 (5 Females)
**Age, years**	54 ± 2	58 ± 3
**BMI, kg/m^2^**	30.8 ± 0.8	31.0 ± 0.92
**Body Fat, %**	39.2 ± 1.8	41.7 ± 4.0
**FPG, mg/dL**	95 ± 4	88 ± 4
**2-h OGT, mg/dL ^§^**	121 ± 12	125 ± 19
**ADA T2DM Risk Assessment Score**	5	5
**Plasma Total Cholesterol, mg/dL**	208 ± 8	212 ± 13
**Plasma LDL Cholesterol, mg/dL**	131 ± 9	124 ± 17
**Plasma HDL Cholesterol, mg/dL**	49 ± 3	56 ± 7
**Plasma Triglycerides, mg/dL**	141 ± 16	160 ± 41

Values are expressed as mean ± standard error of the mean. There were no differences between groups in any characteristics at baseline. Abbreviations: BMI, body mass index; FPG, fasting plasma glucose; OGT, oral glucose tolerance; ADA, American Diabetes Association; LDL, low-density lipoprotein; HDL, high-density lipoprotein; T2DM, type 2 diabetes mellitus. ^§^ placebo group, *n* = 8, and inulin group, *n* = 5.

**Table 3:** Habitual dietary intake of inulin and placebo groups.

	Placebo *n* = 10 (5 Females)	Inulin *n* = 7 (5 Females)
**Energy (kcal/day)**	2172 ± 184	2009 ± 218
**Protein (g/day)**	91 ± 7	74 ± 8 *
**Protein (% kcal/day)**	17 ± 1	15 ± 1
**Carbohydrate (g/day)**	236 ± 19	242 ± 27
**Carbohydrate (% kcal/day)**	44 ± 2	48 ± 3
**Total Fat (g/day)**	94 ± 11	82 ± 13
**Total Fat (% kcal/day)**	38 ± 2	36 ± 2
**Saturated Fat (g/day)**	32 ± 3	27 ± 5
**Saturated Fat (% kcal/day)**	13 ± 1	12 ± 1
**Fiber (g/day)**	17 ± 2	20 ± 2
**Insoluble Fiber (g/day)**	11 ± 1	14 ± 2
**Soluble Fiber (g/day)**	6 ± 1	6 ± 1
**Sodium (mg/day)**	3797 ± 315	2871 ± 248 *
**Betaine (mg/day)**	153 ± 21	187 ± 33
**Choline (mg/day)**	408 ± 32	262 ± 28 **
**l-Carnitine (mg/day)**	51 ± 6	36 ± 3

Values are expressed as mean ± standard error of the mean. * *p* < 0.05 vs. placebo; ** *p* < 0.01 vs. placebo.

## Supplementary Materials

The following are available online at http://www.mdpi.com/2072-6643/10/6/793/s1. Table S1: Composition of standardized diet for the placebo and prebiotic groups, Table S2: Diet composition for each level of energy intake.
