# Supplementary material for: Inulin Supplementation Does Not Reduce Plasma Trimethylamine N-Oxide Concentrations in Individuals at Risk for Type 2 Diabetes
Source: Nutrients. 2018 Jun 20;10(6):793. doi: 10.3390/nu10060793 (PMC6024751; doi:10.3390/nu10060793)
Supplement: Supplementary file 1 [file nutrients-10-00793-s001.zip › TMAO Supplementary Tables.docx]

**Supplemental Table 1. Composition of standardized diet for the placebo and prebiotic groups.**

|  | Placebo | Prebiotic |
| --- | --- | --- |
| Energy (kcal/d) | 2355 ± 147 | 2273 ± 160 |
| Protein (g/d) | 82 ± 5 | 79 ± 6 |
| Carbohydrate (g/d) | 331 ± 21 | 310 ± 19 |
| Total Fat (g/d) | 85 ± 6 | 79 ± 6 |
| Saturated Fat (g/d) | 24 ± 2 | 23 ± 2 |
| Fiber (g/d) § | 13 ± 1 | 13 ± 1 |
| Insoluble Fiber (g/d) | 10 ± 1 | 9 ± 1 |
| Soluble Fiber (g/d) | 4 ± 0 | 4 ± 0 |
| Sodium (mg/d) | 3511 ± 176 | 3500 ± 146 |
| Betaine (mg/d) | 174 ± 9 | 168 ± 10 |
| Choline (mg/d) | 241 ± 12 | 233 ± 13 |
| L-Carnitine (mg/d) | 43 ± 2 | 42 ± 2 |

§ Does not include fiber provided by prebiotic inulin supplement

**Supplemental Table 2. Diet composition for each level of energy intake.**

|  | 1500 | 2000 | 2500 | 3000 |
| --- | --- | --- | --- | --- |
| Energy (kcal/d) | 1432 | 1967 | 2494 | 3051 |
| Protein (g/d) | 54 | 68 | 86 | 105 |
| Carbohydrate (g/d) | 197 | 275 | 345 | 419 |
| Total Fat (g/d) | 50 | 69 | 89 | 110 |
| Saturated Fat (g/d) | 14 | 19 | 25 | 31 |
| Fiber (g/d) § | 9 | 11 | 14 | 17 |
| Insoluble Fiber (g/d) | 6 | 8 | 10 | 13 |
| Soluble Fiber (g/d) | 3 | 3 | 4 | 5 |
| Sodium (mg/d) | 2102 | 3230 | 3670 | 4241 |
| Betaine (mg/d) | 125 | 150 | 180 | 217 |
| Choline (mg/d) | 168 | 208 | 254 | 295 |
| L-Carnitine (mg/d) | 24 | 38 | 45 | 52 |

§ Does not include fiber provided by prebiotic inulin supplement
